# Gut Microbiomes Differ Among Dietary Types and Stool Consistency in the Captive Red Wolf (*Canis rufus*)

**DOI:** 10.3389/fmicb.2020.590212

**Published:** 2020-11-10

**Authors:** Morgan Bragg, Elizabeth W. Freeman, Haw Chuan Lim, Nucharin Songsasen, Carly R. Muletz-Wolz

**Affiliations:** ^1^Department of Environmental Science and Policy, George Mason University, Fairfax, VA, United States; ^2^Center for Species Survival, Smithsonian Conservation Biology Institute, National Zoological Park, Front Royal, VA, United States; ^3^Center for Conservation Genomics, Smithsonian Conservation Biology Institute, National Zoological Park, Washington, DC, United States; ^4^School of Integrative Studies, George Mason University, Fairfax, VA, United States; ^5^Department of Biology, George Mason University, Fairfax, VA, United States

**Keywords:** red wolf, gut microbiome, diet, stool consistency, gastrointestinal health, *Canis rufus*

## Abstract

Captive management of many wildlife species can be challenging, with individuals displaying health disorders that are not generally described in the wild population. Retrospective studies have identified gastrointestinal (GI) diseases, in particular inflammatory bowel disease (IBD), as the second leading cause of captive adult red wolf (*Canis rufus)* mortality. Recent molecular studies show that imbalanced gut microbial composition is tightly linked to IBD in the domestic dog. The goal of the present study was to address two main questions: (1) how do red wolf gut microbiomes differ between animals with loose stool consistency, indicative of GI issues, and those with normal stool consistency and (2) how does dietary type relate to stool consistency and red wolf gut microbiomes? Fresh fecal samples were collected from 48 captive wolves housed in eight facilities in the United States and from two wild wolves living in Alligator River National Wildlife Refuge, NC, United States. For each individual, the stool consistency was categorized as loose or normal using a standardized protocol and their diet was categorized as either wild, whole meat, a mix of whole meat and kibble or kibble. We characterized gut microbiome structure using 16S rRNA gene amplicon sequencing. We found that red wolves with a loose stool consistency differed in composition than wolves with normal stool consistency, suggesting a link between GI health and microbiome composition. Diet was not related to stool consistency but did significantly impact gut microbiome composition; gut microbiome composition of wolves fed a kibble diet were significantly different than the gut microbiome composition of wolves fed a mixed, whole meat and wild diet. Findings from this study increase the understanding of the interplay between diet and GI health in the red wolf, a critical piece of information needed to maintain a healthy red wolf population *ex situ*.

## Introduction

Extreme losses to animal populations globally prompted the establishment of captive breeding programs that strive to maintain genetic diversity and integrity of the species (Association of Zoos and Aquariums Canid Taxonomic Advisory Group, 2012). For example, the American red wolf (*Canis rufus)* is a critically endangered canid that historically inhabited the south eastern United States, specifically stretching from the Atlantic coast to central Texas, with southern New York as the northern barrier down to the Gulf of Mexico (Hinton et al., 2013). The species was nearly exterminated by predator control programs and continues to face threats of hybridization with coyotes and human caused mortality (US Fish and Wildlife Service, 2016). Today, red wolves exist in a captive population and one small reintroduced population in Alligator River National Wildlife Refuge (ARNWR) in North Carolina (US Fish and Wildlife Service, 2016; Phillips, 2018). The *ex situ* population serve to maintain the genetic diversity of the red wolf and is critical for the continued existence of the species (Hedrick and Fredrickson, 2008).

Unfortunately, the captive red wolf population is threatened by health issues, especially gastrointestinal (GI) disease that are not described in the wild population (Acton et al., 2000). Gastrointestinal disease is the second most common cause of mortality in the captive red wolf population (Acton et al., 2000; Seeley et al., 2016). For instance, from 1992 through 2012, 21% (32/151) of mortalities in adult red wolves were related to GI disease, with 25% (8/32) of these individuals suffering from inflammatory bowel disease (IBD) (Acton et al., 2000; Seeley et al., 2016). An additional 25% (37/151) of wolves that died from 1992 to 2012 had non-lethal GI lesions (Acton et al., 2000; Seeley et al., 2016). While IBD did not kill these individuals, 68% (22/37) possessed lesions similar to IBD (Acton et al., 2000; Seeley et al., 2016). Since 2018, there were four GI related deaths within the captive population, one of which was caused by gastric perforations that were potentially associated with IBD (Wolf, 2019).

Currently, the causative agent of red wolf GI disease remains unknown; however, it is probable that complex interactions between GI microbiota, or the bacteria in the gut, environmental factors and a genetic predisposition play a role (Suchodolski et al., 2012a; Henson et al., 2017). A previous study suggests that red wolves may have a genetic predisposition to IBD as the species lacks the putatively protective thymine allele in two single nucleotide polymorphisms (SNP) in the toll-like receptor-5 gene that are associated with IBD in the domestic dog (Henson et al., 2017). While we did not validate the missing putative protective thymine allele in two SNP in the toll-like receptor-5 gene in individuals sampled, we are making the assumption that this missing allele is consistent across all captive red wolves based on the small founder population.

An alteration in the structure of the gut microbiome, or the community of bacteria in the gut, is a common pattern that has been linked to GI diseases in humans and dogs (Suchodolski et al., 2012b; Aziz et al., 2013; Yang et al., 2015; Marchesi et al., 2016; Xu et al., 2016), and may relate to changes in the production of short chain fatty acids (SCFA) by bacterial groups (Parada Venegas et al., 2019). In the GI track of dogs with IBD, studies have shown a relative increase in the phyla Proteobacteria (Minamoto et al., 2015) and Actinobacteria (Xenoulis et al., 2008; Suchodolski, 2011; Gonçalves et al., 2018) and a relative decrease in the phyla Bacteriodetes (Minamoto et al., 2015; Omori et al., 2017) and Fusobacteria (Xenoulis et al., 2008; Suchodolski, 2011; Gonçalves et al., 2018) compared with healthy individuals. Inconsistent results have been found for the phylum Firmicutes, as some studies show an increase (Xenoulis et al., 2008; Suchodolski, 2011; Gonçalves et al., 2018) while others demonstrate a decrease in relative abundance in the gut of domestic dogs with IBD (Suchodolski et al., 2012a; Minamoto et al., 2015). To date, there is no information on the relationship between gut microbial community and GI health in the red wolf.

The increases and decreases in phylum and their association with gut health likely relate to the SCFA being produced such as butyrate by Firmicutes and Fusobacteria (Vital et al., 2014) and acetate and proprionate by Bacteridoetes (Jandhyala et al., 2015). Other bacteria may produce these SCFAs, however, it has not been described. Butyrate can suppress inflammation in immune and epithelial cells by improving pro-inflammatory response of immune cells to antigens (Gonçalves et al., 2018). Butyrate can encourage various properties that benefit intestinal barrier function by coordinating tight junctions that inhibit pro-inflammatory molecules from moving across the gut wall (Chambers et al., 2018) and stoping the build-up of toxic metabolic waste products (Jandhyala et al., 2015). Acetate is the one of the main components that allows certain bacteria to kill pathogens within the GI tract (Ríos-Covián et al., 2016). Additionally, acetate is involved with lipogenesis in fat tissue or oxidized by muscle and this can improve glucose homeostasis and possibly inflammatory status (Chambers et al., 2018). Acetate also can suppress low grade inflammation by reducing the amount of TNF-α, a cytokine involved in inducing and/or maintaining inflammation (van der Beek et al., 2016). The SCFA propionate provides energy to colon epithelial cells (Poeker, 2019), maintains homeostasis in the GI tract (Marchesi et al., 2016) and monitors immune function by triggering an increase in Treg cell production and differentiation (Ríos-Covián et al., 2016), which has anti-inflammatory and anti-metabolic effects. Moreover, propionate is a precursor for glucogenesis in the liver (den Besten et al., 2015) and can induce appetite regulation and reduce food intake in humans (Chambers et al., 2018). As a result of their influential mechanisms, any alteration in presence or concentration of these SCFA can have negative impacts on GI health.

Gut microbiota is linked to diet in humans (Jandhyala et al., 2015), canids (Suchodolski et al., 2012b), and other wild animals (Dhanasiri et al., 2011; Nelson et al., 2013; Amato et al., 2016). In humans, those that eat a diet high in fruits, vegetables and fibers have an increase in the relative abundance of Firmicutes in their gut, while those on a diet high in animal meat have an increase in relative abundance of Bacteroidetes (Jandhyala et al., 2015). In the domestic dog, it has been noted that a high carbohydrate diet can increase the relative abundance of Firmicutes while a high protein diet can increase the relative abundance of Fusobacteria (Hang et al., 2012). Moreover, terrestrial carnivores were documented to have an increase in the relative abundance of Firmicutes and a decrease in the relative abundance of Bacteroidetes (Nelson et al., 2013). Red wolves maintained in human care are commonly fed a diet that includes dry dog kibble, although some individuals are offered commercial meat mixed with their kibble and a small number of wolves receive whole meat or carcass with no kibble. As much as facilities try to mimic the wild diet, these captive diets can be far from the nutritional composition that a wild wolf would get in its natural environment (Association of Zoos and Aquariums Canid Taxonomic Advisory Group, 2012). It has been shown that captive diet can alter the gut microbiome composition and functional diversity of many species housed in zoos (McKenzie et al., 2017). These alterations can cause a decrease in immune function, nutritional uptake, and GI health (McKenzie et al., 2017).

Although histopathological findings of post-mortem tissue have indicated a high prevalence of IBD in the *ex situ* population of red wolves, it is challenging to diagnose IBD in living individuals. The gold standard for IBD diagnosis is endoscopy to assess mucosal surface of the duodenum and obtain full-thickness biopsies for histopathologic evaluation (Seeley et al., 2016; Wolf, 2019). Due to the need for specialized equipment and expertise, which are not readily available in many facilities, stool consistency has been used as a general proxy for GI health in the red wolf (Jergens et al., 2003; Rist et al., 2013; Bermingham et al., 2017; Karu et al., 2018; Falony et al., 2019).

The overall goal of this study was to characterize the gut microbiome of adult captive red wolves and its relationship to GI health. We wanted to answer two main questions with our study (1) how red wolf gut microbiomes differ between individuals with loose stool consistency, which is indicative of GI issues, and normal stool consistency and (2) how dietary type relates to stool consistency and red wolf gut microbiomes. Gastrointestinal diseases, such as IBD are a serious threat to the *ex situ* population of red wolves. There are less than 300 red wolves left in the world, and it is critical to better understand the origin of GI diseases within the captive population.

## Materials and Methods

### Fecal Sample Collection and Scoring

Fecal samples were opportunistically obtained from a total of 50 red wolves, 48 captive wolves housed in eight facilities and two wild wolves (Supplementary Table 1). From captive wolves, a total of 62 fecal samples were collected by keepers during routine physical examinations or within one hour of visual observation of defecation. Samples were kept at −20°C until overnight shipment to the Smithsonian Conservation Biology Institute. Once samples arrived at SCBI, they were stored at −80°C until DNA extraction. Two fecal samples were opportunistically collected from two wild wolves during 2018 trapping efforts of the remaining wild population conducted by US Fish and Wildlife Service in ARNWR in Manteo, North Carolina. The project was exempt due to its opportunistic nature by the Smithsonian National Zoological Park’s IACUC committee and George Mason University’s IACUC committee.

We scored the consistency of each fecal sample once thawed, prior to DNA extraction, based on previously described criteria: 0 = normal/slightly soft feces (*n* = 22), 1 = soft feces with or without blood and/or mucus (*n* = 14), 2 = very soft feces (*n* = 12) and 3 = watery diarrhea (*n* = 1) (Jergens et al., 2003). Samples that were fecal consistency score (FCS) 0 were binned into a “normal” category while FCS 1, 2, and 3 were binned into a “loose stool” category. The loose stool category falls along a spectrum from mild to severe, but we combined the three scores because they each represent a deviation from normal stool consistency. Preliminary statistical analyses indicated that FCS 1 and 2 did not differ from one another in microbiome beta diversity structure (data not shown), providing statistical justification for binning of FCS 1, FCS 2, and FCS 3 together into the loose stool category to increase sample size and include FCS 3 (with a sample size of 1).

### Dietary Types

Individuals were categorized into one of four dietary types: (1) kibble-based diet, (2) a whole meat, (3) mixture of kibble and commercial meat, and (4) wild. Captive wolves were categorized into a group if they were fed that dietary type for at least 5 of the 7 days per week. Kibble-based diets comprised of a high energy, meat-based dry food approved for domestic dogs. Typically, a whole meat diet consisted of donated or roadkill white tail deer, elk, wild turkey, beaver, rats, guinea pig or chicken. Generally, mixed diet consisted of Classic Carnivore Diets—canine or feline meat log (Nebraska brand) comprised largely of horse meat and kibble. Wild red wolves primarily consume white tail deer, rabbit, raccoons, small mammals and various rodents (McVey et al., 2013).

### Molecular Genetics Methods

We extracted DNA from each sample using the QIAamp PowerFecal DNA Kit (#12530-50, Qiagen, MD) following manufacture’s protocol. A sterile 1.5 ml microcentrifuge tube was included for each set of sample extractions as a negative control. We determined DNA concentration and quality on a NanoDrop One (Thermo Fisher Scientific, MA).

We used a two-step polymerase chain reaction (PCR) protocol combined with dual-index paired-end Illumina sequencing to sequence the gut microbiome of each individual. For the first PCR step (amplicon PCR), we amplified a ∼380 base pair region in the V3–V5 region of the 16S rRNA gene using the universal primers 515F (GTGCCAGCMGCCGCGGTAA) and 939R (CTTGTGCGGGCCCCCGTCAATTC). Duplicate PCR reactions were done for each sample and included the negative extraction controls and negative PCR controls. The 20 μl amplicon PCR consisted of 10 μl of 2x Phusion HotStart II HF Master Mix, 1 μM of the forward primer, 1 μM of the reverse primer and 2 μl of DNA template at 10–15 ng/μl concentration. PCR conditions were: (a) activation at 98°C for 30 s, (b) 25 cycles of denaturation at 98°C for 10 s, (c) annealing at 68°C for 20 s, (d) extension at 72°C for 30 s, and (e) a final extension at 72°C for 5 min. We pooled duplicate amplicon PCR together then performed index PCR, attaching custom i5 and i7 adaptors during a second PCR step (index PCR) to provide unique identities to each fecal sample. The 50 μl index PCR assay consisted of 25 μl of 2x Phusion Hot Start II HF Master Mix, 5 μl of the i5 primer, 5μl of the i7 primer and 5 μl of cleaned amplicon products. Index PCR conditions were: (a) activation at 98°C for 2 min, (b) followed by 8 cycles of denaturation at 98°C for 20 s, (c) annealing at 63°C for 30 s, (d) extension at 72°C for 30 s, and (e) a final extension at 72°C for 2 min. We used Speed-beads (in a PEG/NaCl buffer) (Rohland and Reich, 2012) to clean post-PCR products between each PCR reaction and verified PCR products using gel electrophoresis. The concentration of each cleaned index PCR product was measured using a Qubit4 (Invitrogen, MA) and samples were pooled together in equimolar proportion. We ran the pooled library on an E-Gel Power Snap Gel Electrophoresis System (Invitrogen, MA) using a 2% agarose gel cassette and cut out the target band (∼380 basepair). The library from the gel cut was extracted using a QIAquick Gel Extraction Kit (#28704, Qiagen, MD) and diluted to 4 nM. We used real time qPCR, following the KAPA Library Quantification Kit Illumina Platforms protocol, to confirm the concentration of the library (KK4824, Roche Sequencing and Life Sciences, MA) post gel extraction. The pooled library was sequenced on two Illumina MiSeq runs (v3 chemistry: 2 × 300 bp kit) at the Center for Conservation Genomics, National Zoo.

Following sequence generation, we imported demultiplexed reads from the Illumina MiSeq into R version 3.5.0 (R Core Team, 2018). The package “dada2” version 1.12 (Callahan et al., 2016) was used to check for chimeras and filter low-quality sequences (maxEE > 2). We generated amplicon sequence variants (ASVs) and assigned ASV taxonomy by aligning the sequences against the Ribosomal Database Project (RDP) 16S training set 16/release11.5 (Wang et al., 2007). A phylogenetic tree was built in the program Quantitative Insights Into Microbial Ecology 2 (vQIIME2-2018.4) (Bolyen et al., 2018) using the fasttree algorithm (Price et al., 2009). We removed likely contaminant ASVs using the package “decontam” (Davis et al., 2017) and the Fisher method with a threshold of 0.1, which removed three ASVs. We then filtered out any negative control samples and singletons ASVs (ASV that occurs as one sequence in one sample). Some individuals (RW2079, RW2112, RW11559, and RW2247) donated multiple samples and a random number generator was used to choose one sample from each individual to be included in the analyses. One individual (RW2079) donated 11 total samples over a span of 6 months and the overall gut microbiome composition was similar among all samples (Supplementary Figures 2,3), therefore providing justification for including only one sample from each individual. The variation in the sequencing depth was approximately 8x (max = 35,296, min = 4,418) across individuals; therefore, we did not employ normalization correction methods following recommendations by Weiss et al. (2017).

### Statistical Analysis

All statistical analyses were conducted in R version 3.5.0 (R Core Team, 2018) and significance was determined as *p* < 0.05. We used analysis framework based on previous research (Muletz-Wolz et al., 2019a,b) to examine microbiome structure in red wolves. We addressed two main questions through statistical analyses: (1) how do red wolf gut microbiomes differ between loose stool consistency and normal stool consistency and (2) how does dietary type relate to stool consistency and red wolf gut microbiomes? As a general outline for microbiome analysis, we examined variation in microbiome structure at three levels: (1) alpha diversity—which is within-sample variation, (2) beta-diversity—which is between-sample variation, and (3) changes in relative abundance at the phylum and ASV level. For alpha diversity, we examined ASV richness and Faith’s phylogenetic diversity (PD). For beta diversity, we examined Jaccard’s, Bray-Curtis and unweighted Unifrac distances. For relative abundance analyses, we assessed how gut microbiome composition differed between dietary types and stool consistency at the phylum level. To determine if there was an association between stool consistency and dietary type, we conducted a Fisher’s Exact Test using the function *fisher.test*.

We examined if alpha diversity differed among stool consistencies and diet categories using two measures, ASV richness and Faith’s Phylogenetic Diversity (PD). ASV richness is the number of unique bacterial taxa and Faith’s PD estimates how phylogenetically diverse the bacterial community is. We used a Shapiro-Wilk test and a Levene test to verify that our data met the assumptions of normality and homoscedasticity. We conducted an ANOVA with ASV richness or Faith’s PD as the response variable, stool consistency and dietary type as the explanatory variables and facility as the covariate using the “car” package (Fox and Weisberg, 2011). We used the function *TukeyHSD* to perform *post hoc* analyses.

To examine beta diversity, we used PERMANOVAs (Anderson, 2017) with Jaccard, Bray Curtis or unweighted Unifrac distance as the response variable, stool consistency and dietary type as the explanatory variables and facility as the covariate in the package “vegan” (Oksanen et al., 2019). Facility accounted for 19–21% of the variation in the data set (Supplementary Figure 1) so we wanted to account for it, however, we wanted to focus on dietary type and stool consistency. Jaccard distances are based on presence-absence of bacterial taxa, Bray-Curtis is abundance-weighted and Unifrac is presence-absence while accounting for phylogenetic relationships. We examined the dispersion of the microbial communities using PERMDISP to determine if community variance differed among dietary types and stool consistency, separately.

We used two methods to assess bacterial taxa that differed between stool consistency and among dietary types. First, we used the package “DAtest” to identify the best statistical tests to highlight bacterial taxa that are common between stool consistency categories and among dietary types in two separate analyses (Russel et al., 2018). We used the function *preDA* to filter out non-prevalent ASVs that were present in less than 12 samples for dietary type and less than 10 samples for stool consistency. We chose these parameters because it removed ASVs that did not have enough shared information among samples but retained the rare ASVs of potential interest (Russel et al., 2018). For both stool consistency and dietary type, differential abundance testing was performed at the ASV level to examine if there were any specific ASVs that were common among categories. Additionally, differential abundance testing at the phylum level was assessed because this taxonomic level has often been examined in canid IBD literature (Kim et al., 2017; Pilla and Suchodolski, 2020). We then input raw sequence counts of the filtered ASV table or unfiltered phylum level table. The *testDA* function was used to perform the various default transformations of the data based on the statistical test. The three differential abundance tests used had the lowest False Positive Rate for our data at the ASV level (Russel et al., 2018). We only reported ASVs or phyla that were significant in at least two out of the three of the top ranked differential abundance tests. For dietary type, the statistical tests used were an ANOVA—Multiplicative zero-correction and additive log-ration normalization (function *DA.aoa)*, Kruskal-Wallis test (function *DA.kru)* and linear regression with multiplicative zero-correction and center log-ration normalization (function *DA.lmc)*. For stool consistency, the statistical tests used were Quasi-Poisson general linear model (function *DA.qpo)*, Wilcox Rank Sum and Signed Rank Test (function *DA.wil)* and Welch *t*-test (function *DA.ltt*). Additionally, indicator species analysis using the package “indicspecies” (De Caceres and Legendre, 2009) was performed to uncover any patterns of relative abundance at the ASV level associated with dietary types or stool consistency. The function *multipatt* was used on a relative abundance matrix. An ASV was considered a valid indicator species if the *p*-value was less than 0.05 and the indicator value was 0.5 or greater (De Cáceres et al., 2012). An indicator value of 1 means that the ASV is found in all samples from one group and completely absent from the other group (De Cáceres et al., 2012). On the contrary, an indicator value of 0 means that the ASV was commonly found in samples from both groups (De Cáceres et al., 2012). The same species matrix was used for both methods of differential abundance testing. Each method comes with its own assumptions and limitations; using both gives more insight into taxa associated with stool consistency and diet categories, specifically the indicator values in the indicator species analysis.

## Results

We characterized the gut microbiome of 50 wolves from 8 facilities (Supplementary Table 1). Samples from 22 wolves had normal stool consistency and 28 wolves had loose stool consistency (Table 1). Thirty-four wolves were categorized as kibble diet, three wolves under whole meat diet, 10 wolves under the mixed diet and two wolves under the wild dietary type (Table 1). There was no association between stool consistency and dietary type (Fisher’s Exact Test, *p* = 0.2198).

**TABLE 1:** Number of wolves in each stool consistency category and dietary type category.

	**Kibble**	**Mixed**	**Meat**	**Wild**	**Stool consistency total**
Normal stool consistency	16	4	0	2	22
Loose stool consistency	19	5	3	0	28
Diet total	35	10	3	2	

*Stool consistency was used as a proxy to estimate GI health. The dietary type categories were composed of kibble, mixed kibble and meat, whole meat and wild dietary type.*

**FIGURE 1:**
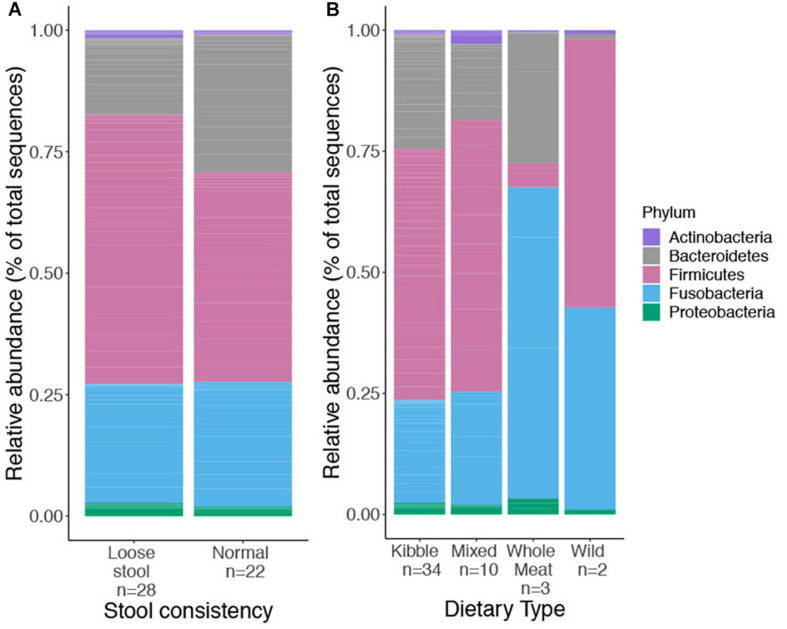
Relative abundance of top bacterial phyla found in the gut microbiome of captive and wild red wolves as it related to stool consistency categories **(A)**, used as a proxy for GI health and four dietary types **(B)**; kibble, mixed, whole meat or wild dietary type. No phyla differed significantly in relative abundance between stool consistency or dietary types.

We obtained 729,356 high quality sequences from 50 red wolf fecal samples (mean = 14,693 sequences, min = 4,418, max = 35,296). A total of 436 ASVs, belonging to five bacteria phyla, Bacteroidetes, Firmicutes, Proteobacteria, Actinobacteria, and Fusobacteria, were identified. We examined relative abundance of each phylum among the four dietary types and stool consistency, respectively (Figure 1).

### Gut Microbiota Relative to Stool Consistency

**FIGURE 2:**
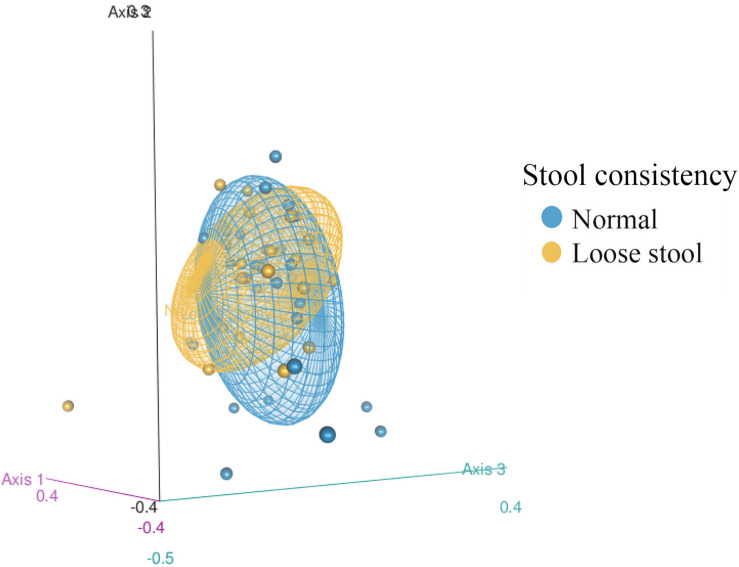
3D Non-metric multidimensional scaling (NMDS) of fecal bacterial community structure from 50 red wolves (unweighted Unifrac distance matrix) between stool consistency categories. 95% confidence ellipses shown for stool consistency categories, loose and normal.

Gut microbiome structure of both stool consistency categories were similar in the number of taxa present and phylogenetic diversity (ASV richness ANOVA: *F*_stat_ = 0.946, *p* = 0.337, *df* = 1; Faith’s PD ANOVA: *F*_stat_ = 1.498, *p* = 0.229, *df* = 1), but differed in composition (Figure 2, PERMANOVA: Jaccard Pseudo-*F* = 1.7644, *df* = 1, *R*^2^ = 3%, *p* = 0.01; Bray Pseudo-*F* = 1.7373, *df* = 1, *R*^2^ = 2.9%, *p* = 0.055; unweighted Unifrac Pseudo-*F* = 1.8595, *df* = 1, *R*^2^ = 2.97%, *p* = 0.017). There was similar dispersion in the gut bacterial community between stool consistency categories (PERMDISP: Jaccard *p* = 0.2889, Bray-Curtis *p* = 0.3628, unweighted Unifrac *p* = 0.2008).

**FIGURE 3:**
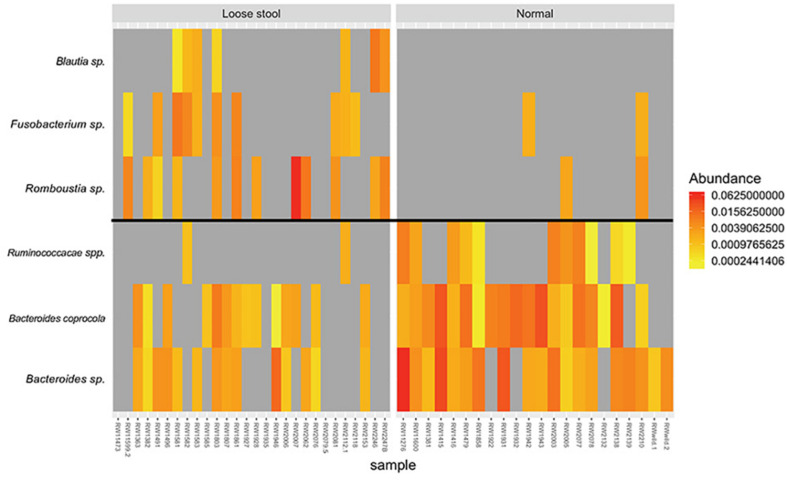
Heatmap of relative abundance differences in bacterial taxa between the gut microbiome of red wolves with loose **(left group)** and normal stool consistency **(right group)**. All taxa identified by indicator species analysis.

There were no ASVs or bacterial phyla that were identified by differential abundance analysis between stool consistency categories. Indicator species analysis identified three ASVs associated with loose stool consistency: *Blautia* sp. (*p* = 0.022, indicator value = 0.5), *Romboutsia* sp. (*p* = 0.01, indicator value = 0.623), and *Fusobacterium* sp. (*p* = 0.019, indicator value = 0.569) (Figure 3). Three ASVs were found to be associated with normal stool consistency: *Bacteroides caprocola* (*p* = 0.001, indicator value = 0.863), *Bacteroides* sp. (*p* = 0.001, indicator value = 0.859), and *Ruminococcacae* spp. (*p* = 0.002, indicator value = 0.693) (Figure 3).

### Gut Microbiota Relative to Dietary Type

**FIGURE 4:**
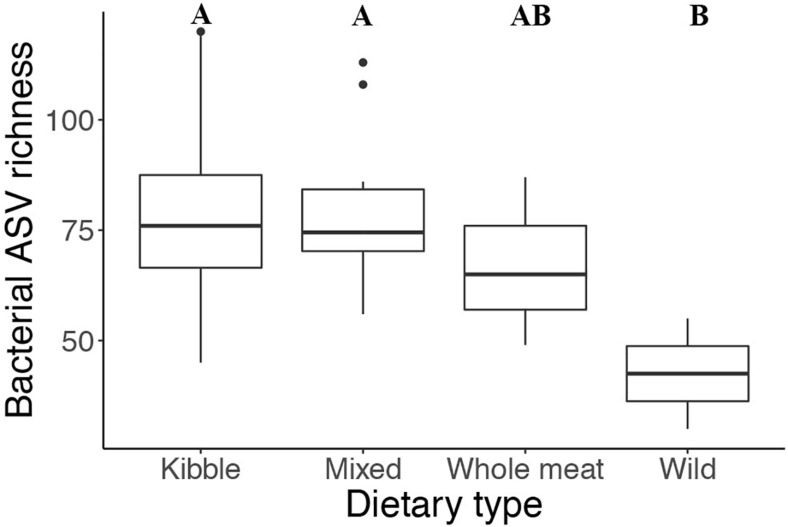
Gut bacterial taxa richness in the four different dietary types of the captive and wild red wolf. Wild red wolf gut microbiome had lower bacterial taxa richness than the gut microbiome of captive red wolves fed kibble and mixed diets (superscripts: Tukey’s HSD: wild—kibble *p* = 0.02, wild—mixed *p* = 0.03).

Dietary type influenced the number of bacteria present and the phylogenetic diversity of the gut microbiota. Gut bacterial ASV richness was significantly different among the four dietary types (ANOVA: *F*_stat_ = 3.142, *p* = 0.04, *df* = 3) (Figure 4), with individuals consuming a wild diet having a lower number of bacterial taxa than those consuming a kibble and mixed diet (TukeyHSD: wild—kibble *p* = 0.02, wild—mixed *p* = 0.03). Phylogenetic diversity (Faith’s PD) also differed among dietary types (ANOVA: *F*_stat_ = 5.053, *p* = 0.005, *df* = 3), with the wild individuals having lower Faith’s PD compared to the kibble diet, mixed diet and whole meat diets (TukeyHSD: wild—kibble *p* = 0.02, wild—mixed *p* = 0.004, wild—whole meat *p* = 0.01).

**FIGURE 5:**
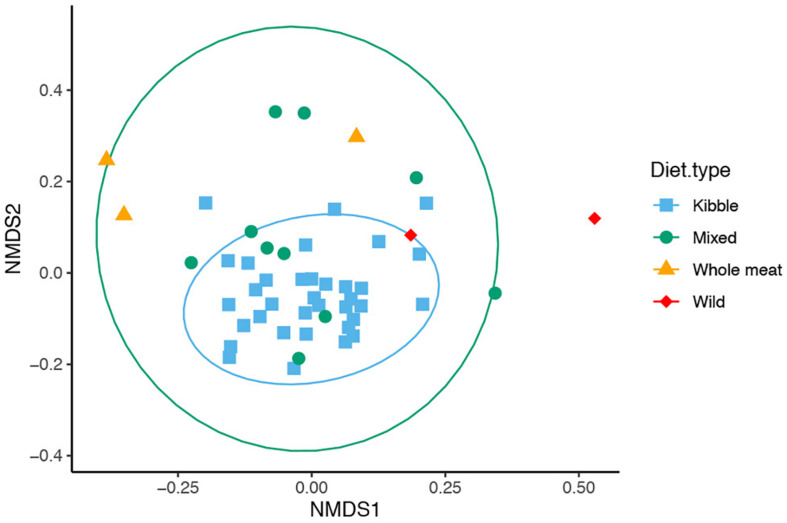
Non-metric multidimensional scaling (NMDS) of gut microbiome composition of 50 red wolves sorted by dietary types (unweighted Unifrac distance matrix). Specific differences in overall gut microbiome composition were seen between kibble and mixed diet, mixed and whole meat diet, kibble and whole meat diet and wild and kibble diets. 95% confidence ellipses shown for dietary types that had greater than 3 samples.

We found that dietary type influenced bacterial community composition in the red wolf GI tract (Figure 5, PERMANOVA: Jaccard Psuedo-*F* = 2.518, *df* = 3, *R*^2^ = 12.9%, *p* = 0.001; Bray Psuedo-*F* = 2.1454, *df* = 3, *R*^2^ = 10.9%, *p* value = 0.001; unweighted Unifrac Psuedo-*F* = 3.3257, *df* = 3, *R*^2^ = 15.9%, *p* = 0.001). Gut bacterial community of wolves that were offered a kibble diet differed from that of individuals who consumed a whole meat diet (pairwise *p* < 0.05 for Bray-Curtis, Jaccard, Unifrac distance), mixed diet (pairwise *p* < 0.05 for Jaccard and Unifrac distance) and wild diet (pairwise *p* < 0.05 for Jaccard and Unifrac distance). Wolves that consumed a mixed diet also differed from whole meat diet (pairwise *p* < 0.05 for Jaccard and Unifrac distance). The bacterial communities had dissimilar dispersion (PERMDISP: Jaccard *p* = 0.01, Bray-Curtis *p* = 0.01), which may be driving the significant difference in bacterial community among the dietary types.

**FIGURE 6:**
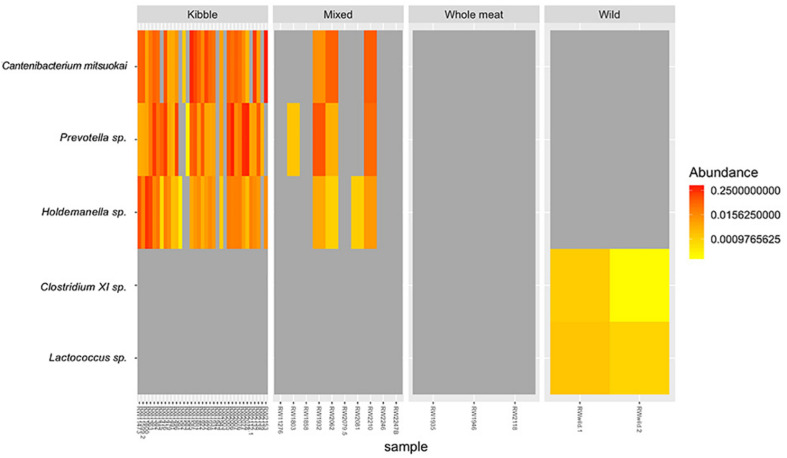
Relative abundance of bacterial taxa that differed in the gut microbiome of red wolves fed the four dietary types; kibble, mixed, whole meat and wild diet. Taxa identified by indicator species analysis and/or DA.test.

We found three bacterial ASVs that differed in relative abundance among dietary types, particularly between kibble diet and other dietary types, but no variation in relative abundances was detected at the phylum level. The relative abundance of ASV *Catenibacterium mitsuokai* (*DA.aoa p* = 0.003; *DA.lmc p* = 0.01), *Holdemanella* sp. (*DA.aoa p* = 0.003; *DA.lmc p* = 0.001; *DA.kru p* = 0.04), and *Prevotella* sp. (*DA.aoa p* = 0.004; *DA.lma p* = 0.004) were significantly higher in wolves fed a kibble diet compared to all other dietary types (Figure 6). Indicator species analysis identified one ASV associated with kibble dietary type: *Holdemanella* sp. (*p* = 0.029, indicator value = 0.901) (Figure 6). Two ASVs identified with the wild dietary type: *Clostridium XI* sp. (*p* = 0.002, indicator value = 1) and *Lactococcus* sp. (*p* = 0.002, indicator value = 1).

## Discussion

The gut is filled with a large population of bacteria that protect against pathogens, ferment non-digestible dietary carbohydrates and aid in the development of the immune system (Omori et al., 2017). Diet can impact the presence and/or abundance of bacterial taxa, which can negatively impact canid GI health (Suchodolski et al., 2012b; McKenzie et al., 2017). This study was the first to characterize the gut microbiome composition of the red wolf, a critically endangered canid, and its relationship with diet and stool consistency. Bacterial phyla, and their relative abundances, in red wolf gut microbiomes were similar to those observed in other mammals (Nishida and Ochman, 2018), including canids (Zhang and Chen, 2010; Deng and Swanson, 2015; Suchodolski, 2016; US Fish and Wildlife Service, 2016; Alessandri et al., 2019a). Specifically, bacterial phyla Firmicutes, Bacteroidetes and Fusobacteria are the most abundant in the canid gut, including the red wolf.

We found that stool consistency is associated with gut microbial composition as also detected in other studies (Vandeputte et al., 2016; Müller et al., 2020). In the present study, gut microbiome composition differed between captive red wolves with normal versus loose stool consistency, suggesting that GI health is likely tied to microbiome composition. We detected a significant increase in the relative abundance *Blautia* sp., *Fusobacterium* sp., and *Romboutsia* sp. in the gut microbiome of red wolves with loose stool in comparison to red wolves with normal stool consistency. *Blautia* sp. (phylum Firmicutes) is associated with Crohn’s disease (Tedjo et al., 2016), irritable bowel syndrome with diarrhea (Craven et al., 2017) and softer stool (Müller et al., 2020) in human. Additionally, a relative increase in *Blautia* sp. is associated with an increase in the number of lymphocytes and white blood cells in weaned piglets (Zhu et al., 2018b). Bacteria in the phylum Firmicutes produce the SCFA butyrate which can impact immune response to inflammation (Corthay, 2009; Gonçalves et al., 2018). A relative increase in this phylum could lead to an excess in butyrate production which could induce inflammation, leading improper immune response (Ratajczak et al., 2019). A relative increase of bacterial taxa in the *Fusobacterium* genus (phylum Fusobacteria) is associated with periodontal disease and Crohn’s disease in humans (Allen-Vercoe et al., 2011). Although periodontal disease and Crohn’s disease are in different locations of the body these diseases share a common characteristic; oversensitivity to mutualistic bacteria (Allen-Vercoe et al., 2011). Bacteria in the phylum Firmicutes produce the SCFA butyrate which also can influence the ability of the host’s immune system to recognize commensal bacteria (Gonçalves et al., 2018). A relative increase in abundance of this genus could alter the ability to properly recognize commensal bacteria in the gut. The genus *Romboutsia* (phylum Firmicutes) is a taxa commonly seen in the mammalian gut, including the dog. Although these bacteria produce SCFAs, like acetate and lactate (Gerritsen, 2015), their roles in maintaining GI health are relatively unknown (Gerritsen et al., 2019).

We detected a significant increase in the relative abundance of the family *Ruminococcacae, Bacteroides coprocola*, and *Bacteroides* sp. in the gut of wolves with normal stool consistency. The family *Ruminococcacae* (phylum Firmicutes) is associated with the production of butyrate via carbohydrate fermentation in the human gut (Bamberger et al., 2018). The genus *Bacteroides* (phylum Bacteroidetes) is commonly found in the human gut; those with a healthy gut have a higher relative abundance in their gut than individuals suffering from IBD (Miyake et al., 2015). It is possible that bacterial taxa associated with loose stool consistency could be triggering an immune response or that the bacterial taxa associated with normal stool consistency could be maintaining healthy gut function in the captive red wolf, or both.

Studies in domestic cat and dog demonstrate a link between dietary type and stool quality (Suchodolski et al., 2012b). In the present study, we did not detect a similar relationship between diet and stool consistency in captive red wolves. Other factors, including ingestion of inappropriate food items (Gómez-Gallego et al., 2016), appearance of a specific pathogens (Suchodolski et al., 2012b), intestinal parasites disease and tumors or mechanical obstruction (Dandrieux, 2016) also could impact stool quality. The presence of diarrhea can be sporadic in canids (Gómez-Gallego et al., 2016), and establishing the link between stool quality and diet may require longitudinal samples collected over time. Furthermore, stool consistency is only one proxy for GI health. Future studies should combine other metrics, like body condition score, serum cobalamin and serum folate concentration, to identify wolves with poor GI health. Low serum cobalamin and folate concentrations are documented in canids with GI issues (Songsasen and Rodden, 2010). Having an altered gut microbiome and loose stool consistency could indicate an alteration in nutrient and water intake due to increased transit time in the GI tract (Vandeputte et al., 2016).

We demonstrated that changes in the gut microbiome structure was linked to diet in the red wolf. The gut microbiome of captive wolves that ate the kibble diet was generally different than all other dietary types, and particularly different from of captive individuals offered a whole meat diet. We expected the gut microbiome of wolves eating a whole meat diet to have similar composition to the gut microbiome of wild wolves, however, analysis revealed that composition was not analogous. While the wolves in both diet categories eat similar species, it is possible that the difference in gut microbiome composition is driven by variations in the proportion of the primary food sources (white tail deer), secondary food sources (small mammals), and tertiary food sources (rodents) consumed. Fluctuations in the ratio of prey eaten is evident across the various wild wolf packs, with differences driven by location of the pack territory (Phillips et al., 2003; Dellinger et al., 2011). Additionally, Dellinger et al. (2011) reported wild red wolves consuming anthropogenic waste from a garbage dump and carcass pit, another possibility for the variation in gut microbiome composition seen. Wolves that were offered the kibble diet had an increase in the relative abundance of the phylum Firmicutes while individuals that were fed the whole meat diet had an increase in the relative abundance of the phylum Fusobacteria. The phylum Firmicutes is associated with a high carbohydrate diet whereas the phylum Fusobacteria is associated with a high protein diet in domestic dogs (Hang et al., 2012). The kibble-based diet contains a large amount of starch (30–60% of dry matter) (Fortes et al., 2010), which likely increases the relative abundance of Firmicutes in the gut microbiome of red wolves in the present study. Similarly, the whole meat dietary type is rich in protein, and this likely explained the increases in the abundance of Fusobacteria in the gut microbiome of wolves eating this diet. Variation in relative abundance of these phyla in the gut could impact the concentration of SCFA produced (den Besten et al., 2013).

Diet can reinforce intestinal barrier function and structure via SCFA production (Parada Venegas et al., 2019). However, if gut microbiome structure is altered by diet then it can impact the amount of SCFA by products produced (Ratajczak et al., 2019). This shift in SCFA production could be because of a change in presence or relative abundance of SCFA producing bacteria or the down regulation of G protein coupled receptors (GPCR) (Parada Venegas et al., 2019). These SCFAs and GPCRs work together to maintain intestinal barrier integrity by inducing mucosal healing and suppressing inflammation (Parada Venegas et al., 2019). Furthermore, there is evidence of diet impacting GPCR activity, specifically the expression of GPR43 is reduced in the intestinal mucosa of mice eating a high fat/sugar diet (Parada Venegas et al., 2019). It is possible that diet is shifting the gut microbiome composition of captive red wolves, thus changing the concentrations of these SCFAs. While butyrate, acetate and propionate have beneficial impacts on immune function and intestinal barrier integrity, there also could be negative impacts, like inflammation, from having sustained elevated levels of these SCFAs (Ohira et al., 2017; Ratajczak et al., 2019). Additionally, too much or too little of SCFAs can lead to obesity or other metabolic syndromes in humans and mice (Ohira et al., 2017; Ratajczak et al., 2019). It is possible that there needs to be an optimal level of each SCFA for beneficial impacts to occur in the gut of the red wolf.

We found that there was an increase in the relative abundance of the bacterial taxa *Holdemanella* sp., *Cantenibacterium mitsuokai*, and *Prevotella* sp. in kibble diets compared to all other dietary types. *Holdemanella* sp. (phylum Firmicutes) produce butyrate, acetic and propionic acid (De Maesschalck et al., 2014) and are associated with gut microbiome variations triggered by a diet high in fat and sugars in humans (Dubé et al., 2018). *Cantenibacterium mitsuokai* (phylum Firmicutes) is linked to gut microbiome alterations triggered by a high fat and sugar diets in humans (Dubé et al., 2018) and with carbohydrates fermentation and propionate production in felids (Butowski et al., 2019). Similarly, *Prevotella* sp. (phylum Bacteroidetes) are correlated with kibble diets, carbohydrate fermentation and propionate production in felids (Butowski et al., 2019) and domestic dogs (Bermingham et al., 2017). Humans and mice with irritable bowel syndrome suffering from recurrent diarrhea show an increase in bacteria from this genus (Su et al., 2018). We documented two bacteria taxa only in the gut microbiome of the wild wolves that were not found in captive wolves; *Clostridium XI* sp., *Lactococcus* sp., and *Clostridium XI* sp. (phylum Firmicutes) are found in the gut microbiome of healthy domestic cats and dogs (Handl et al., 2011; Pilla and Suchodolski, 2020) and involved in breakdown of bile acid, digestion of dietary fats and fat-soluble vitamins (Deng and Swanson, 2015). Additionally, this taxa prompts anti-inflammatory reactions in the gut of the domestic dogs (Pilla and Suchodolski, 2020). *Lactococcus* sp. (phylum Firmicutes) has been identified in the gut of other canids, including the Artic wolf and coyote (Finlayson-Trick et al., 2017), and certain strains of this taxa are used to cultivate probiotic dairy products for humans (Cook et al., 2018). It is possible that bacterial taxa associated with kibble diets are present to aid in the digestion of the dietary material but could also be triggering GI inflammation due to their foreign nature and/or production of SCFAs at chronic elevated levels in the captive red wolf.

For reintroductions of red wolves to be successful, it is imperative that the captive red wolves are kept as “wild” as possible, including their gut microbiome. The gut microbiome needs time to acclimate to the wild environment during reintroductions (Yao et al., 2019). If animals are not given proper time to fully acclimate their gut microbiome to the wild environment, it could increase their susceptibility to pathogens, decrease survival rates, impact proper nutrient transformation, vitamin production and communication between the microbiota and the immune system (Weiss and Hennet, 2017; Yao et al., 2019). We found that bacterial richness in the wild wolves’ gut microbiome was significantly lower compared to the captive wolves’ gut microbiome. Contact between humans and wolves in a captive setting could account for the higher bacterial richness in the gut of the captive wolves; it is possible for microbes to transfer from humans to animals and vice versa while living in close proximity to each other (Trinh et al., 2018). We also found higher abundances of Bacteroidetes and lower abundances of Fusobacteria in captive animals compared to wild wolves, which is seen in other mammals globally (McKenzie et al., 2017) and in semi-wild wolves in China (Wu et al., 2017). These alterations caused by captivity and diet difference can change the overall functional diversity of the gut microbiota, in turn impacting GI function. A non-natural diet can attract non-natural bacteria or alter the relative abundance of natural gut flora (Henson et al., 2017; Alessandri et al., 2019b).

Our strongest finding with implications to management was that kibble-based diets was linked to the most distinct gut microbiome, suggesting that the approach to diet may need to be reconsidered. While the kibble diet is nutritionally complete for the domestic dog, it does contain a high amount of starch (Fortes et al., 2010). Although the domestic dog and the wolf are closely related, there are still evolutionary differences that separate the two species. There is evidence that the domestication of the dog from the wolf was marked by an increase in the ability to digest a starch rich diet (Axelsson et al., 2013). Wolves possess significantly lower number of diploid copy numbers and expression of the AMY2B gene, which encodes for amylase expression in the pancreas, allowing the breakdown of starch, than the domestic dog (Axelsson et al., 2013). Furthermore, Zhu et al. (2018) reported that wolves are adapted to a meat-based diet implying that meat should be incorporated into their daily diet. Based off this evidence, it is clear that the wolf may not be as adapted to this starch rich diet as the domestic dog. Therefore, the approach to diet may need to be reconsidered so that the daily diet of the captive red wolf does include whole meat.

Future studies that would help address the link between diet, GI health and the microbiome could monitor the gut microbiome alongside incorporating dietary changes. For example, whole meat could be added into the daily diet of a wolf eating purely kibble while simultaneously assessing the gut microbiome to monitor composition shifts toward the wild wolf gut microbiome structure. Additional wild red wolf samples would increase the understanding of the wild gut microbiome structure. If captive red wolves were to be released into the wild, it may be necessary to closely mimic the gut microbiome composition of the wild red wolf prior to release.

In conclusion, we characterized the gut microbiome of the red wolf, a critically endangered canid, that is suffering greatly from GI health issues. We found that gut microbiome structure was influenced by stool consistency and dietary type. Wolves consuming a wild dietary type had lower bacterial richness and phylogenetic diversity than their captive counterparts, suggesting that captivity may be introducing novel bacteria into the captive red wolf gut microbiome—altering it from its wild state. It is possible that captive red wolves are acquiring bacteria taxa because of their functional role in digesting captive diets, however, these bacteria may not be beneficial for the red wolf. There are approximately 280 red wolves left in the world, with 95% of individuals living in captivity. While the captive population is necessary for the continued existence of the species, the captive environment brings manipulated diets and man-made structures to live in (Williams et al., 2019). These factors, along with others, can cause shifts in the gut microbiome (Williams et al., 2019), negatively impacting GI health of the red wolf. It is essential for the captive population to be healthy to facilitate successful reintroductions of these animals into their native landscape in the future.

## Data Availability Statement

The datasets presented in this study can be found in online repositories. The names of the repository/repositories and accession number(s) can be found below: https://www.ncbi.nlm.nih.gov/, PRJNA647909.

## Ethics Statement

The animal study was reviewed and approved by the IACUC—George Mason University and IACUC—Smithsonian Institution.

## Author Contributions

MB, EF, NS, and CM-W planned the study and the experimental design. MB and NS facilitated sample collection from facilities. MB performed DNA extraction, amplification library preparation, and sequencing and wrote the manuscript. CM-W provided guidance to MB in laboratory methods. MB and CM-W performed analysis and interpreted results. EF, NS, HL, and CM-W edited the manuscript. All authors contributed to the article and approved the submitted version.

## Conflict of Interest

The authors declare that the research was conducted in the absence of any commercial or financial relationships that could be construed as a potential conflict of interest.

## References

[B1] Acton A. E. Munson L. Waddell W. T. (2000). Survey of necropsy results In captive red wolves (Canis Rufus), 1992-1996. *J. Zoo Wildl. Med.* 31 2–8. 10.1638/1042-7260(2000)031[0002:sonric]2.0.co;2 10884116

[B2] Alessandri G. Milani C. Mancabelli L. Mangifesta M. Lugli G. A. Viappiani A. (2019a). Metagenomic dissection of the canine gut microbiota: insights into taxonomic, metabolic and nutritional features. *Environ. Microbiol.* 21 1331–1343. 10.1111/1462-2920.14540 30680877

[B3] Alessandri G. Milani C. Mancabelli L. Mangifesta M. Lugli G. A. Viappiani A. (2019b). The impact of human-facilitated selection on the gut microbiota of domesticated mammals. *FEMS Microbiol. Ecol.* 95:fiz121. 10.1093/femsec/fiz121 31344227

[B4] Allen-Vercoe E. Strauss J. Chadee K. (2011). *Fusobacterium nucleatum*: An emerging gut pathogen? *Gut Microbes* 2 294–298. 10.4161/gmic.2.5.18603 22067936

[B5] Amato K. R. Metcalf J. L. Song S. J. Hale V. L. Clayton J. Ackermann G. (2016). Using the gut microbiota as a novel tool for examining colobine primate GI health. *Glob. Ecol. Conserv.* 7 225–237. 10.1016/j.gecco.2016.06.004

[B6] Anderson M. J. (2017). “Permutational Multivariate Analysis of Variance (PERMANOVA),” in *Wiley StatsRef: Statistics Reference Online*, ed. StatsRef W. (Hoboken, NY: John Wiley & Sons), 1–15. 10.1002/9781118445112.stat07841

[B7] Association of Zoos and Aquariums Canid Taxonomic Advisory Group. (2012). *Large Canid (Canidae) Care Manual.* Silver Spring, MD: Association of Zoos and Aquariums.

[B8] Axelsson E. Ratnakumar A. Arendt M.-L. Maqbool K. Webster M. T. Perloski M. (2013). The genomic signature of dog domestication reveals adaptation to a starch-rich diet. *Nature* 495 360–364. 10.1038/nature11837 23354050

[B9] Aziz Q. Doré J. Emmanuel A. Guarner F. Quigley E. M. M. (2013). Gut microbiota and gastrointestinal health: current concepts and future directions. *Neurogastroenterol. Motil.* 25 4–15. 10.1111/nmo.12046 23279728

[B10] Bamberger C. Rossmeier A. Lechner K. Wu L. Waldmann E. Fischer S. (2018). A walnut-enriched diet affects gut microbiome in healthy caucasian subjects: a randomized, controlled trial. *Nutrients* 10:244. 10.3390/nu10020244 29470389 PMC5852820

[B11] Bermingham E. N. Maclean P. Thomas D. G. Cave N. J. Young W. (2017). Key bacterial families (Clostridiaceae, Erysipelotrichaceae and Bacteroidaceae) are related to the digestion of protein and energy in dogs. *PeerJ* 5:e3019. 10.7717/peerj.3019 28265505 PMC5337088

[B12] Bolyen E. Rideout J. R. Dillon M. R. Bokulich N. A. Abnet C. Al-Ghalith G. A. (2018). QIIME 2: reproducible, interactive, scalable, and extensible microbiome data science. *PeerJ Inc* 6:e27295v2 10.7287/peerj.preprints.27295v2 PMC7015180 31341288

[B13] Butowski C. F. Thomas D. G. Young W. Cave N. J. McKenzie C. M. Rosendale D. I. (2019). Addition of plant dietary fibre to a raw red meat high protein, high fat diet, alters the faecal bacteriome and organic acid profiles of the domestic cat (Felis catus). *PLoS One* 14:e0216072. 10.1371/journal.pone.0216072 31042730 PMC6493751

[B14] Callahan B. J. McMurdie P. J. Rosen M. J. Han A. W. Johnson A. J. A. Holmes S. P. (2016). DADA2: high-resolution sample inference from Illumina amplicon data. *Nat. Methods* 13 581–583. 10.1038/nmeth.3869 27214047 PMC4927377

[B15] Chambers E. S. Preston T. Frost G. Morrison D. J. (2018). Role of gut microbiota-generated short-chain fatty acids in metabolic and cardiovascular health. *Curr. Nutr. Rep.* 7 198–206. 10.1007/s13668-018-0248-8 30264354 PMC6244749

[B16] Cook D. P. Gysemans C. Mathieu C. (2018). Lactococcus lactis as a versatile vehicle for tolerogenic immunotherapy. *Front. Immunol.* 8:1961. 10.3389/fimmu.2017.01961 29387056 PMC5776164

[B17] Core Team R. (2018). *R: A language and environment for statistical computing.* Vienna: R Foundation for Statistical Computing.

[B18] Corthay A. (2009). How do regulatory T cells work? *Scand. J. Immunol.* 70 326–336. 10.1111/j.1365-3083.2009.02308.x 19751267 PMC2784904

[B19] Craven L. J. Silverman M. Burton J. P. (2017). Transfer of altered behaviour and irritable bowel syndrome with diarrhea (IBS-D) through fecal microbiota transplant in mouse model indicates need for stricter donor screening criteria. *Ann. Transl. Med.* 5:490. 10.21037/atm.2017.10.03 29299452 PMC5750285

[B20] Dandrieux J. R. S. (2016). Inflammatory bowel disease versus chronic enteropathy in dogs: are they one and the same? *J. Small. Anim. Pract.* 57 589–599. 10.1111/jsap.12588 27747868

[B21] Davis N. M. Proctor D. Holmes S. P. Relman D. A. Callahan B. J. (2017). Simple statistical identification and removal of contaminant sequences in marker-gene and metagenomics data. *Microbiome* 6:226. 10.1186/s40168-018-0605-2 PMC6298009 30558668

[B22] De Caceres M. Legendre P. (2009). Associations between species and groups of sites: indices and statistical inference. *Ecology* 90 3566–3574. 10.1890/08-1823.1 20120823

[B23] De Cáceres M. Legendre P. Wiser S. K. Brotons L. (2012). Using species combinations in indicator value analyses. *Methods Ecol. Evol.* 3 973–982. 10.1111/j.2041-210X.2012.00246.x

[B24] De Maesschalck C. Van Immerseel F. Eeckhaut V. De Baere S. Cnockaert M. Croubels S. (2014). Faecalicoccus acidiformans gen. nov., sp. nov., isolated from the chicken caecum, and reclassification of Streptococcus pleomorphus (Barnes et al. 1977), Eubacterium biforme (Eggerth 1935) and Eubacterium cylindroides (Cato et al. 1974) as Faecalicoccus pleomorphus comb. nov., Holdemanella biformis gen. nov., comb. nov. and Faecalitalea cylindroides gen. nov., comb. nov., respectively, within the family Erysipelotrichaceae. *Int. J. Syst. Evol. Microbiol.* 64 3877–3884. 10.1099/ijs.0.064626-0 25180093

[B25] Dellinger J. A. Ortman B. L. Steury T. D. Bohling J. Waits L. P. (2011). Food Habits of Red Wolves during Pup-Rearing Season. *Southeast. Nat.* 10 731–740. 10.1656/058.010.0412 22708719

[B26] den Besten G. Bleeker A. Gerding A. van Eunen K. Havinga R. van Dijk T. H. (2015). Short-Chain fatty acids protect against high-fat diet-induced obesity via a PPARγ-Dependent Switch From Lipogenesis to Fat Oxidation. *Diabetes* 64 2398–2408. 10.2337/db14-1213 25695945

[B27] den Besten G. van Eunen K. Groen A. K. Venema K. Reijngoud D.-J. Bakker B. M. (2013). The role of short-chain fatty acids in the interplay between diet, gut microbiota, and host energy metabolism. *J. Lipid Res.* 54 2325–2340. 10.1194/jlr.R036012 23821742 PMC3735932

[B28] Deng P. Swanson K. S. (2015). Gut microbiota of humans, dogs and cats: current knowledge and future opportunities and challenges. *Br. J. Nutr.* 113 S6–S17. 10.1017/S0007114514002943 25414978

[B29] Dhanasiri A. K. S. Brunvold L. Brinchmann M. F. Korsnes K. Bergh Ø Kiron V. (2011). Changes in the intestinal microbiota of wild atlantic cod (*Gadus morhua*) L. upon captive rearing. *Microb. Ecol.* 61 20–30. 10.1007/s00248-010-9673-y 20424834

[B30] Dubé M. P. Park S. Y. Ross H. Love T. M. T. Morris S. R. Lee H. Y. (2018). Daily HIV pre-exposure prophylaxis (PrEP) with tenofovir disoproxil fumarate-emtricitabine reduced Streptococcus and increased Erysipelotrichaceae in rectal microbiota. *Sci. Rep.* 8:15212. 10.1038/s41598-018-33524-6 30315206 PMC6185988

[B31] Falony G. Vandeputte D. Caenepeel C. Vieira-Silva S. Daryoush T. Vermeire S. (2019). The human microbiome in health and disease: hype or hope. *Acta. Clin. Belg.* 74 53–64. 10.1080/17843286.2019.1583782 30810508

[B32] Finlayson-Trick E. C. L. Getz L. J. Slaine P. D. Thornbury M. Lamoureux E. Cook J. (2017). Taxonomic differences of gut microbiomes drive cellulolytic enzymatic potential within hind-gut fermenting mammals. *PLoS One* 12:e0189404. 10.1371/journal.pone.0189404 29281673 PMC5744928

[B33] Fortes C. M. L. S. Carciofi A. C. Sakomura N. K. Kawauchi I. M. Vasconcellos R. S. (2010). Digestibility and metabolizable energy of some carbohydrate sources for dogs. *Anim. Feed Sci. Tech.* 156 121–125. 10.1016/j.anifeedsci.2010.01.009

[B34] Fox J. Weisberg S. (2011). *An {R} Companion to Applied Regression, Second Edition.* Thousand Oaks, CA: Sage.

[B35] Gerritsen J. (2015). *The genus Romboutsia: Genomic And Functional Characterization of Novel Bacteria Dedicated to Life in The Intestinal Tract.* Wageningen: Wageningen University.

[B36] Gerritsen J. Hornung B. Ritari J. Paulin L. Rijkers G. T. Schaap P. J. (2019). A comparative and functional genomics analysis of the genus Romboutsia provides insight into adaptation to an intestinal lifestyle. *bioRxiv [Preprint]* 10.1101/845511

[B37] Gómez-Gallego C. Junnila J. Männikkö S. Hämeenoja P. Valtonen E. Salminen S. (2016). A canine-specific probiotic product in treating acute or intermittent diarrhea in dogs: a double-blind placebo-controlled efficacy study. *Vet. Microbiol.* 197 122–128. 10.1016/j.vetmic.2016.11.015 27938673

[B38] Gonçalves P. Araújo J. R. Di Santo P. J. (2018). A cross-talk between microbiota-derived short-chain fatty acids and the host mucosal immune system regulates intestinal homeostasis and inflammatory bowel disease. *Inflamm. Bowel Dis.* 24 558–572. 10.1093/ibd/izx029 29462379

[B39] Handl S. Dowd S. E. Garcia-Mazcorro J. F. Steiner J. M. Suchodolski J. S. (2011). Massive parallel 16S rRNA gene pyrosequencing reveals highly diverse fecal bacterial and fungal communities in healthy dogs and cats. *FEMS Microbiol. Ecol.* 76 301–310. 10.1111/j.1574-6941.2011.01058.x 21261668

[B40] Hang I. Rinttila T. Zentek J. Kettunen A. Alaja S. Apajalahti J. (2012). Effect of high contents of dietary animal-derived protein or carbohydrates on canine faecal microbiota. *BMC Vet. Res.* 8:90. 10.1186/1746-6148-8-90 22735212 PMC3464166

[B41] Hedrick P. W. Fredrickson R. J. (2008). Captive breeding and the reintroduction of Mexican and red wolves. *Mol. Ecol.* 17 344–350. 10.1111/j.1365-294X.2007.03400.x 18173506

[B42] Henson L. H. Songsasen N. Waddell W. Wolf K. N. Emmons L. Gonzalez S. (2017). Characterization of genetic variation and basis of inflammatory bowel disease in the Toll-like receptor 5 gene of the red wolf and the maned wolf. *Endanger. Species Res.* 32 135–144. 10.3354/esr00790

[B43] Hinton J. W. Chamberlain M. J. Rabon D. R. (2013). Red wolf (*Canis rufus*) recovery: a review with suggestions for future research. *Animals* 3 722–744. 10.3390/ani3030722 26479530 PMC4494459

[B44] Jandhyala S. M. Talukdar R. Subramanyam C. Vuyyuru H. Sasikala M. Reddy D. N. (2015). Role of the normal gut microbiota. *World. J. Gastroenterol.* 21 8787–8803. 10.3748/wjg.v21.i29.8787 26269668 PMC4528021

[B45] Jergens A. E. Schreiner C. A. Frank D. E. Niyo Y. Ahrens F. E. Eckersall P. D. (2003). A scoring index for disease activity in canine inflammatory bowel disease. *J. Vet. Intern. Med.* 17 291–297. 10.1111/j.1939-1676.2003.tb02450.x 12774968

[B46] Karu N. Deng L. Slae M. Guo A. C. Sajed T. Huynh H. (2018). A review on human fecal metabolomics: methods, applications and the human fecal metabolome database. *Anal. Chim. Acta* 1030 1–24. 10.1016/j.aca.2018.05.031 30032758

[B47] Kim J. An J.-U. Kim W. Lee S. Cho S. (2017). Differences in the gut microbiota of dogs (*Canis lupus familiaris*) fed a natural diet or a commercial feed revealed by the Illumina MiSeq platform. *Gut Pathogens* 9:68. 10.1186/s13099-017-0218-5 29201150 PMC5697093

[B48] Marchesi J. R. Adams D. H. Fava F. Hermes G. D. A. Hirschfield G. M. Hold G. (2016). The gut microbiota and host health: a new clinical frontier. *Gut* 65 330–339. 10.1136/gutjnl-2015-309990 26338727 PMC4752653

[B49] McKenzie V. J. Song S. J. Delsuc F. Prest T. L. Oliverio A. M. Korpita T. M. (2017). The effects of captivity on the mammalian gut microbiome. *Integr. Comp. Biol.* 57 690–704. 10.1093/icb/icx090 28985326 PMC5978021

[B50] McVey J. M. Cobb D. T. Powell R. A. Stoskopf M. K. Bohling J. H. Waits L. P. (2013). Diets of sympatric red wolves and coyotes in northeastern North Carolina. *J. Mammal* 94 1141–1148. 10.1644/13-MAMM-A-109.1

[B51] Minamoto Y. Otoni C. C. Steelman S. M. Büyükleblebici O. Steiner J. M. Jergens A. E. (2015). Alteration of the fecal microbiota and serum metabolite profiles in dogs with idiopathic inflammatory bowel disease. *Gut Microbes* 6 33–47. 10.1080/19490976.2014.997612 25531678 PMC4615558

[B52] Miyake S. Kim S. Suda W. Oshima K. Nakamura M. Matsuoka T. (2015). Dysbiosis in the gut microbiota of patients with multiple sclerosis, with a striking depletion of species belonging to clostridia XIVa and IV clusters. *PLoS One* 10:e0137429. 10.1371/journal.pone.0137429 26367776 PMC4569432

[B53] Muletz-Wolz C. R. Fleischer R. C. Lips K. R. (2019a). Fungal disease and temperature alter skin microbiome structure in an experimental salamander system. *Mol. Ecol.* 28 2917–2931. 10.1111/mec.15122 31066947

[B54] Muletz-Wolz C. R. Kurata N. P. Himschoot E. A. Wenker E. S. Quinn E. A. Hinde K. (2019b). Diversity and temporal dynamics of primate milk microbiomes. *Am. J. Primatol.* 81:e22994. 10.1002/ajp.22994 31219214 PMC6842035

[B55] Müller M. Hermes G. D. A. Canfora E. E. Smidt H. Masclee A. A. M. Zoetendal E. G. (2020). Distal colonic transit is linked to gut microbiota diversity and microbial fermentation in humans with slow colonic transit. *Am. J. of Physiol. Gastrointest. Liver Physiol.* 318 G361–G369. 10.1152/ajpgi.00283.2019 31869241

[B56] Nelson T. M. Rogers T. L. Carlini A. R. Brown M. V. (2013). Diet and phylogeny shape the gut microbiota of Antarctic seals: a comparison of wild and captive animals. *Environ. Microbiol.* 15 1132–1145. 10.1111/1462-2920.12022 23145888

[B57] Nishida A. H. Ochman H. (2018). Rates of gut microbiome divergence in mammals. *Mol. Ecol.* 27 1884–1897. 10.1111/mec.14473 29290090 PMC5935551

[B58] Ohira H. Tsutsui W. Fujioka Y. (2017). Are short chain fatty acids in gut microbiota defensive players for inflammation and atherosclerosis? *J. Atheroscler. Thromb.* 24 660–672. 10.5551/jat.RV17006 28552897 PMC5517538

[B59] Oksanen J. Guillaume Blanchet F. Friendly M. Kindt R. Legendre P. McGlinn D. (2019). *Vegan: Community Ecology Package. R package version 2.5-5.* Available online at: https://CRAN.R-project.org/package=vegan (accessed September 1,2019).

[B60] Omori M. Maeda S. Igarashi H. Ohno K. Sakai K. Yonezawa T. (2017). Fecal microbiome in dogs with inflammatory bowel disease and intestinal lymphoma. *J. Vet. Med. Sci.* 79 1840–1847. 10.1292/jvms.17-0045 28993566 PMC5709562

[B61] Parada Venegas D. De la Fuente M. K. Landskron G. González M. J. Quera R. Dijkstra G. (2019). Short chain fatty acids (SCFAs)-mediated gut epithelial and immune regulation and its relevance for inflammatory bowel diseases. *Front. Immunol* 10:277. 10.3389/fimmu.2019.00277 30915065 PMC6421268

[B62] Phillips M. (2018). *Canis rufus. The IUCN Red List of Threatened Species 2018: e.T3747A119741683.* Available online at: 10.2305/IUCN.UK.2018-2.RLTS.T3747A119741683.en. (Accessed October, 2019)

[B63] Phillips M. K. Henry V. G. Kelly B. T. (2003). *Restoration of the red wolf in wolves: behavior, ecology and conservation.* Chicago, IL: University of Chicago Press, 272–288.

[B64] Pilla R. Suchodolski J. S. (2020). The role of the canine gut microbiome and metabolome in health and gastrointestinal disease. *Front. Vet. Sci.* 6:498. 10.3389/fvets.2019.00498 31993446 PMC6971114

[B65] Poeker S. A. T. M. (2019). *Towards Understanding The Modulation Potential Of Dietary Fibers On Intestinal Microbiota Using Human And A Novel Murine Intestinal Fermentation Model.* Zurich: ETH Zurich Doctoral thesis.

[B66] Price M. N. Dehal P. S. Arkin A. P. (2009). FastTree: computing large minimum evolution trees with profiles instead of a distance matrix. *Mol. Biol. Evol.* 26 1641–1650. 10.1093/molbev/msp077 19377059 PMC2693737

[B67] Ratajczak W. Rył A. Mizerski A. Walczakiewicz K. Sipak O. Laszczyńska M. (2019). Immunomodulatory potential of gut microbiome-derived short-chain fatty acids (SCFAs). *Acta Biochim. Pol.* 66 1–12. 10.18388/abp.2018_2648 30831575

[B68] Ríos-Covián D. Ruas-Madiedo P. Margolles A. Gueimonde M. de los Reyes-Gavilán C. G. Salazar N. (2016). Intestinal short chain fatty acids and their link with diet and human health. *Front. Microbiol.* 7:185. 10.3389/fmicb.2016.00185 26925050 PMC4756104

[B69] Rist V. T. S. Weiss E. Eklund M. Mosenthin R. (2013). Impact of dietary protein on microbiota composition and activity in the gastrointestinal tract of piglets in relation to gut health: a review. *Animal* 7 1067–1078. 10.1017/S1751731113000062 23410993

[B70] Rohland N. Reich D. (2012). Cost-effective, high-throughput DNA sequencing libraries for multiplexed target capture. *Genome Res.* 22 939–946. 10.1101/gr.128124.111 22267522 PMC3337438

[B71] Russel J. Thorsen J. Brejnrod A. D. Bisgaard H. Sorensen S. Burmolle M. (2018). DAtest: a framework for choosing differential abundance or expression method. *bioRxiv[Preprint]* 10.1101/241802

[B72] Seeley K. E. Garner M. M. Waddell W. T. Wolf K. N. (2016). A survey of diseases in captive red wolves (Canis rufus), 1997–2012. *J. Zoo Wildlife Med.* 47 83–90. 10.1638/2014-0198.1 27010267

[B73] Songsasen N. Rodden M. D. (2010). The role of the Species Survival Plan in maned wolf (*Chrysocyon brachyurus*) conservation. *Int. Zoo Yb.* 44 136–148. 10.1111/j.1748-1090.2009.00093.x

[B74] Su T. Liu R. Lee A. Long Y. Du L. Lai S. (2018). Altered intestinal microbiota with increased abundance of Prevotella is associated with high risk of diarrhea-predominant irritable bowel syndrome. *Gastroent. Res. Pract.* 2018:6961783. 10.1155/2018/6961783 29967640 PMC6008816

[B75] Suchodolski J. S. (2011). Companion animal symposium: Microbes and gastrointestinal health of dogs and cats. *J. Anim. Sci.* 89 1520–1530. 10.2527/jas.2010-3377 21075970 PMC7199667

[B76] Suchodolski J. S. (2016). Diagnosis and interpretation of intestinal dysbiosis in dogs and cats. *Vet. J.* 215 30–37. 10.1016/j.tvjl.2016.04.011 27160005

[B77] Suchodolski J. S. Dowd S. E. Wilke V. Steiner J. M. Jergens A. E. (2012a). 16S rRNA gene pyrosequencing reveals bacterial dysbiosis in the duodenum of dogs with idiopathic inflammatory bowel disease. *PLoS One* 7:e39333. 10.1371/journal.pone.0039333 22720094 PMC3376104

[B78] Suchodolski J. S. Markel M. E. Garcia-Mazcorro J. F. Unterer S. Heilmann R. M. Dowd S. E. (2012b). The fecal microbiome in dogs with acute diarrhea and idiopathic inflammatory bowel disease. *PLoS One* 7:e51907. 10.1371/journal.pone.0051907 23300577 PMC3530590

[B79] Tedjo D. I. Smolinska A. Savelkoul P. H. Masclee A. A. van Schooten F. J. Pierik M. J. (2016). The fecal microbiota as a biomarker for disease activity in Crohn’s disease. *Sci. Rep.* 6:35216. 10.1038/srep35216 27734914 PMC5062155

[B80] Trinh P. Zaneveld J. R. Safranek S. Rabinowitz P. M. (2018). One health relationships between human, animal, and environmental microbiomes: a mini-review. *Front. Public Health* 6:235. 10.3389/fpubh.2018.00235 30214898 PMC6125393

[B81] US Fish and Wildlife Service (2016). *History of the Red Wolf Recovery Program.* Available online at: https://www.fws.gov/redwolf/redwolfrecovery.html (accessed October 2019)

[B82] van der Beek C. M. Canfora E. E. Lenaerts K. Troost F. J. Olde Damink S. W. M. Holst J. J. (2016). Distal, not proximal, colonic acetate infusions promote fat oxidation and improve metabolic markers in overweight/obese men. *Clin. Sci. (Lond).* 130 2073–2082. 10.1042/CS20160263 27439969

[B83] Vandeputte D. Falony G. Vieira-Silva S. Tito R. Y. Joossens M. Raes J. (2016). Stool consistency is strongly associated with gut microbiota richness and composition, enterotypes and bacterial growth rates. *Gut* 65 57–62. 10.1136/gutjnl-2015-309618 26069274 PMC4717365

[B84] Vital M. Howe A. C. Tiedje J. M. (2014). Revealing the bacterial butyrate synthesis pathways by analyzing (Meta)genomic Data. *mBio* 5:14. 10.1128/mBio.00889-14 24757212 PMC3994512

[B85] Wang Q. Garrity G. M. Tiedje J. M. Cole J. R. (2007). Naïve Bayesian classifier for rapid assignment of rRNA sequences into the new bacterial taxonomy. *Appl. Environ. Microbiol.* 73 5261–5267. 10.1128/AEM.00062-07 17586664 PMC1950982

[B86] Weiss G. A. Hennet T. (2017). Mechanism and consequences of intestinal dysbiosis. *Cell. Mol. Life Sci.* 74 2959–2977. 10.1007/s00018-017-2509-x 28352996 PMC11107543

[B87] Weiss S. Xu Z. Z. Peddada S. Amir A. Bittinger K. Gonzalez A. (2017). Normalization and microbial differential abundance strategies depend upon data characteristics. *Microbiome* 5:27. 10.1186/s40168-017-0237-y 28253908 PMC5335496

[B88] Williams C. L. Caraballo-Rodríguez A. M. Allaband C. Zarrinpar A. Knight R. Gauglitz J. M. (2019). Wildlife-microbiome interactions and disease: exploring opportunities for disease mitigation across ecological scales. *Drug Discov. Today: Dis. Models* 28 105–115. 10.1016/j.ddmod.2019.08.012

[B89] Wolf K. (2019). “Vet update on SSP population,” in *Proceedings of the Red Wolf Species Survival Plan meeting* (Albany, GA).

[B90] Wu X. Zhang H. Chen J. Shang S. Yan J. Chen Y. (2017). Analysis and comparison of the wolf microbiome under different environmental factors using three different data of Next Generation Sequencing. *Sci. Rep.* 7:11332. 10.1038/s41598-017-11770-4 28900198 PMC5596057

[B91] Xenoulis P. G. Palculict B. Allenspach K. Steiner J. M. House V. M. A. Suchodolski J. S. (2008). Molecular-phylogenetic characterization of microbial communities imbalances in the small intestine of dogs with inflammatory bowel disease. *FEMS Microbiol. Ecol.* 66 579–589. 10.1111/j.1574-6941.2008.00556.x 18647355

[B92] Xu J. Verbrugghe A. Lourenço M. Janssens G. P. J. Liu D. J. X. Van de Wiele T. (2016). Does canine inflammatory bowel disease influence gut microbial profile and host metabolism? *BMC Vet. Res.* 12:114. 10.1186/s12917-016-0736-2 27306031 PMC4910228

[B93] Yang T. Santisteban M. M. Rodriguez V. Li E. Ahmari N. Carvajal J. M. (2015). Gut dysbiosis is linked to hypertension novelty and significance. *Hypertension* 65 1331–1340. 10.1161/HYPERTENSIONAHA.115.05315 25870193 PMC4433416

[B94] Yao R. Xu L. Hu T. Chen H. Qi D. Gu X. (2019). The “wildness” of the giant panda gut microbiome and its relevance to effective translocation. *Glob. Ecol. Conserv* 18:e00644 10.1016/j.gecco.2019.e00644

[B95] Zhang H. Chen L. (2010). Phylogenetic analysis of 16S rRNA gene sequences reveals distal gut bacterial diversity in wild wolves (*Canis lupus*). *Mol. Biol. Rep.* 37 4013–4022. 10.1007/s11033-010-0060-z 20306230

[B96] Zhu J. Gao M. Song X. Zhao L. Li Y. Hao Z. (2018b). Changes in bacterial diversity and composition in the faeces and colon of weaned piglets after feeding fermented soybean meal. *J. Med. Microbiol.* 67 1181–1190. 10.1099/jmm.0.000766 29923819

[B97] Zhu L. Wu Q. Deng C. Zhang M. Zhang C. Chen H. (2018). Adaptive evolution to a high purine and fat diet of carnivorans revealed by gut microbiomes and host genomes. *Environ. Microbiol.* 20 1711–1722. 10.1111/1462-2920.14096 29528548

