# Supplementary material for: Gut Microbiomes Differ Among Dietary Types and Stool Consistency in the Captive Red Wolf (Canis rufus)
Source: Front Microbiol. 2020 Nov 10;11:590212. doi: 10.3389/fmicb.2020.590212 (PMC7693430; doi:10.3389/fmicb.2020.590212)
Supplement: Supplementary file 1 [file Data_Sheet_1.docx]

***Supplementary Material***

**Gut microbiomes differ among dietary types and stool consistency in the captive red wolf (*Canis rufus)***

Morgan Bragg^1,2,5*^, Elizabeth W. Freeman^3^, Haw Chuan Lim^4^, Nucharin Songsasen^5^, Carly R. Muletz-Wolz^2^

*Correspondence:

Morgan Bragg

[mbragg2@masonlive.gmu.edu](mailto:mbragg2@masonlive.gmu.edu)

**Supplementary Figures**


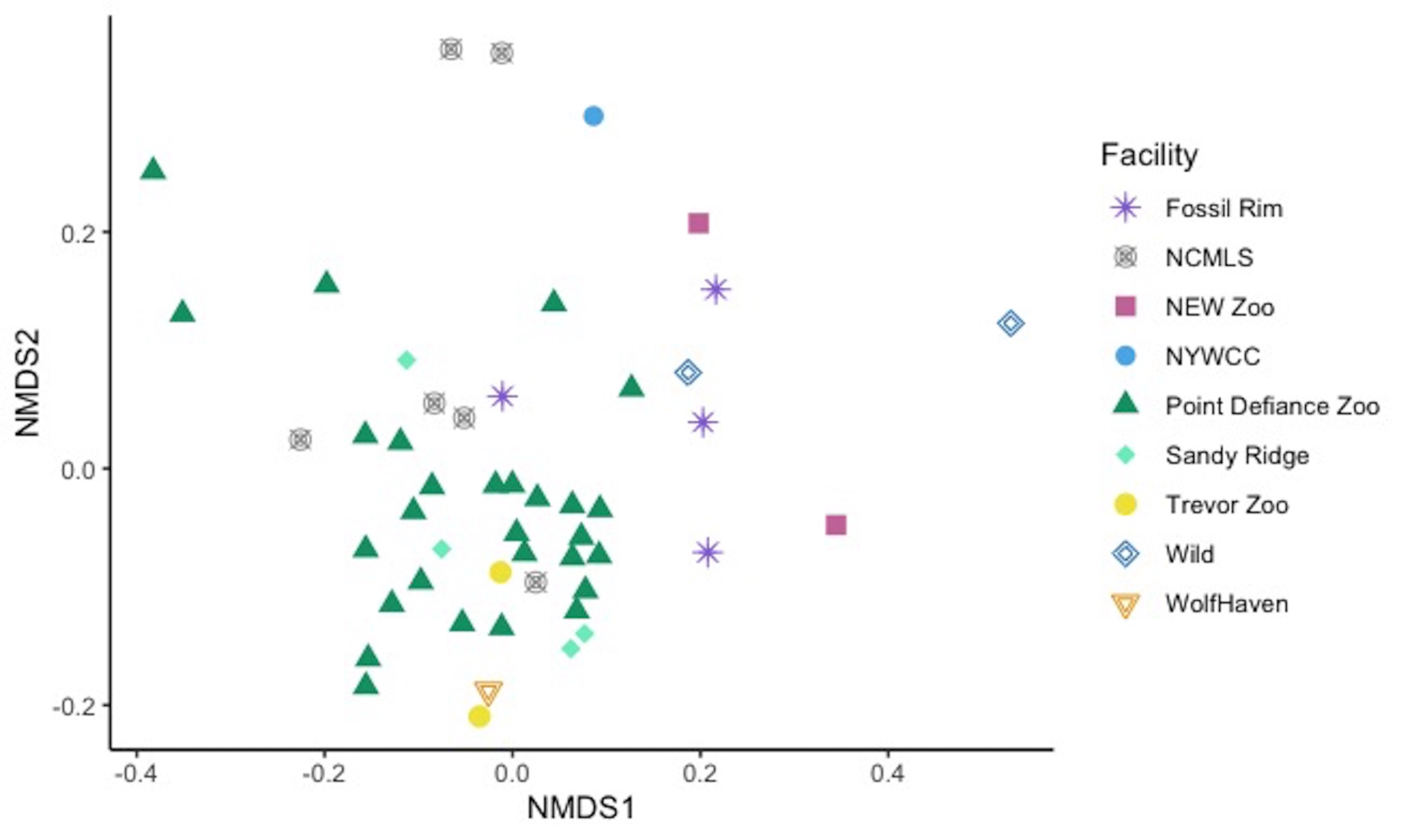


**Supplementary Figure 1.** Non-metric multidimensional scaling **(**NMDS) of fecal bacterial community structure from 50 red wolves (unweighted Unifrac distance matrix) from eight different facilities.

**
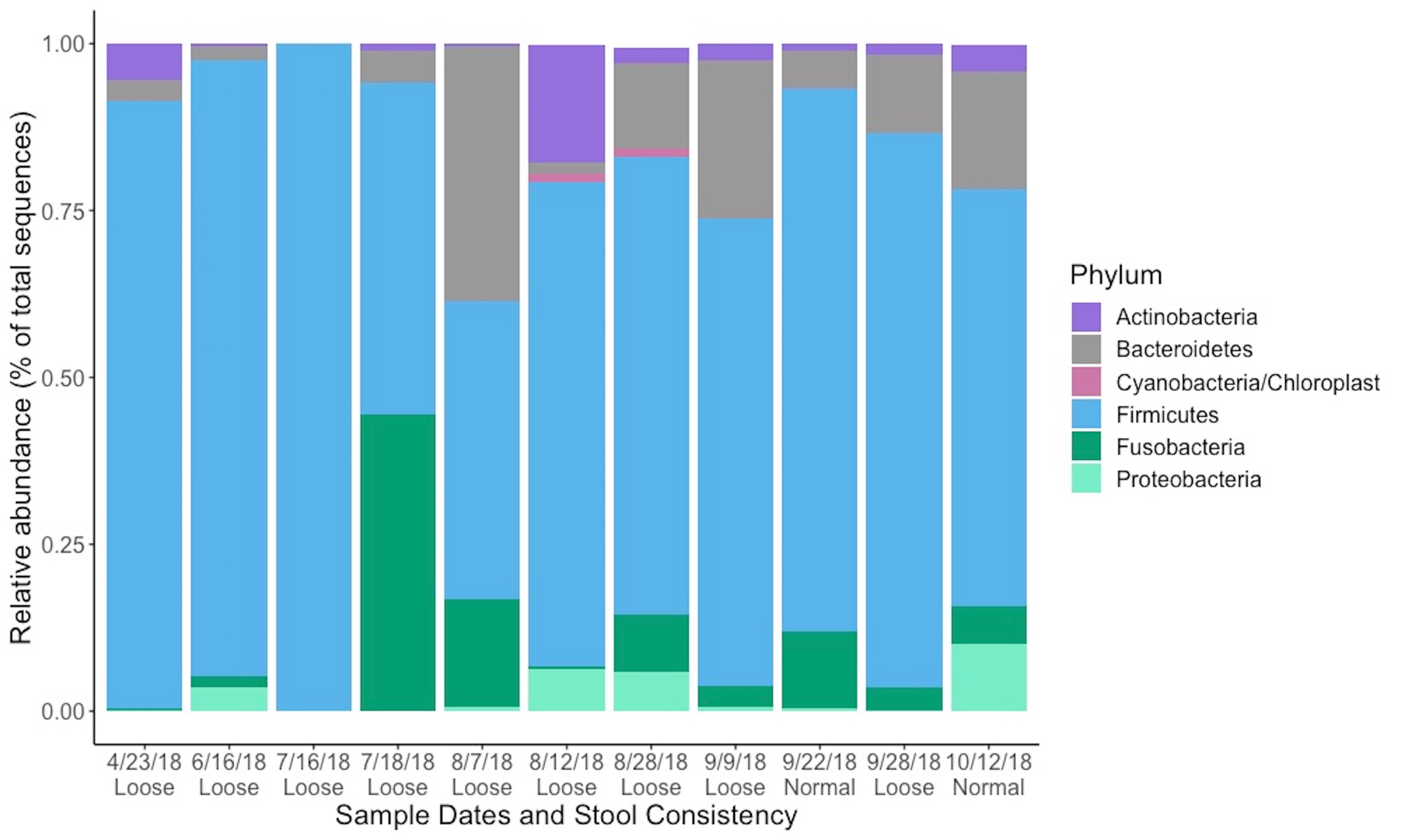
**

**Supplementary Figure 2.** Comparison of relative abundance stacked bar plots of top bacterial phyla found in the gut microbiome of red wolf, RW2079, across 11 samples over the time span of six months. Ten samples were categorized as loose stool consistency and two samples were categorized as normal stool consistency.


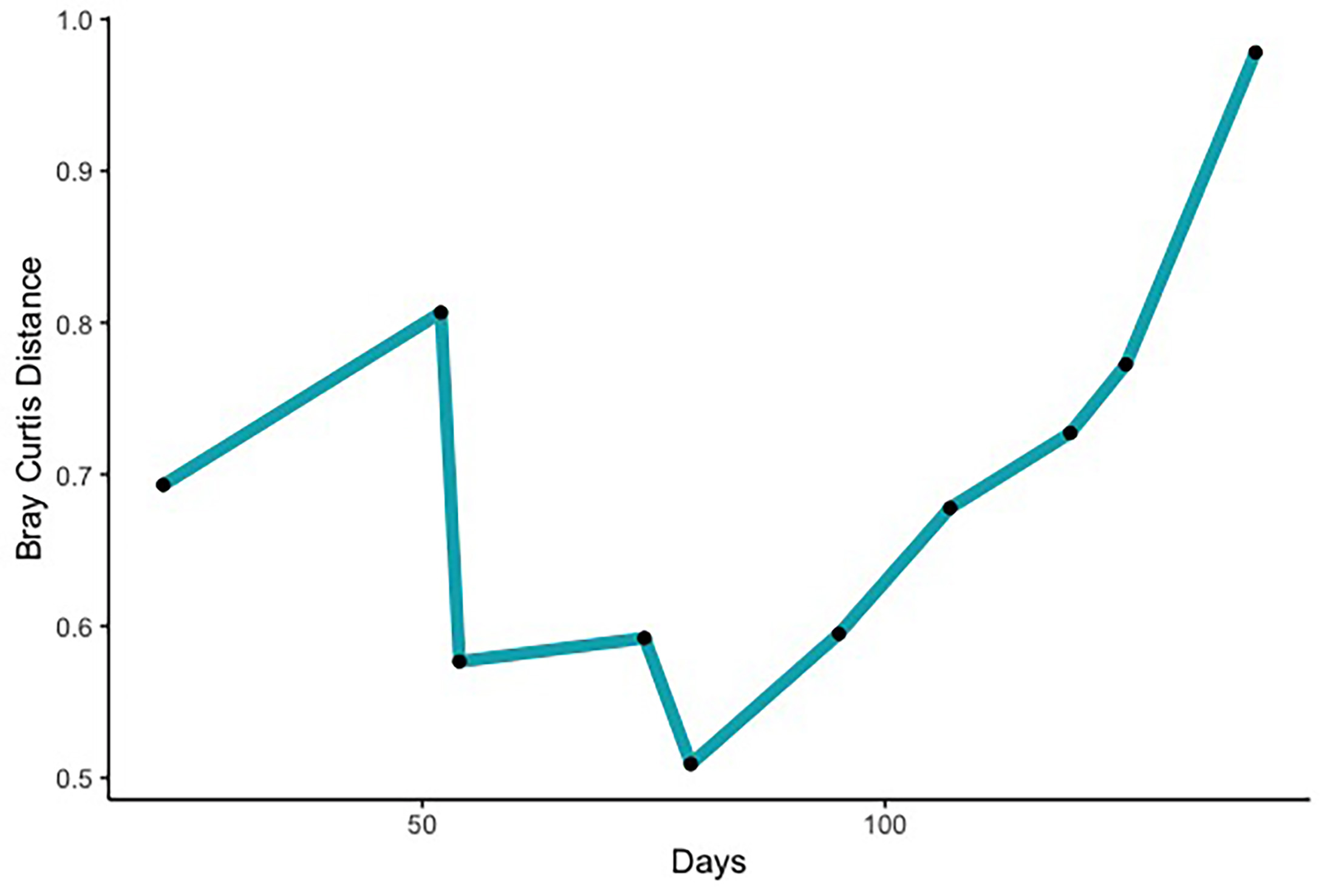


**Supplementary Figure 3.**  Longitudinal change in Bray-Curtis distances between successive samples collected from red wolf, RW2079. There are a total of 11 samples over the time span of six months.

**Supplementary Table**

**Data Sheet 1.** List of each participating wolf, the SSP facility and its location, the age, sex and number of samples collected from each wolf and their dietary type. Total number of samples is 67.

| **Studbook number** | **Facility** | **Location** | **Age** | **Sex** | **# of samples** | **Dietary type** |
| --- | --- | --- | --- | --- | --- | --- |
| 1582 | Fossil Rim Wildlife Center | Glen Rose, TX | 10 | F | 1 | Kibble |
| 1583 | Fossil Rim Wildlife Center | Glen Rose, TX | 10 | F | 1 | Kibble |
| 1581 | Fossil Rim Wildlife Center | Glen Rose, TX | 11 | M | 1 | Kibble |
| 2112 | Fossil Rim Wildlife Center | Glen Rose, TX | 3 | F | 2 | Kibble |
| 2118 | New York Wolf Conservation Center | Salem, NY | 2 | M | 1 | Whole meat |
| 1858 | North Carolina Museum of Life and Science | Durham, NC | 6 | F | 1 | Mixed |
| 2210 | North Carolina Museum of Life and Science | Durham, NC | 6 months | F | 1 | Mixed |
| 1803 | North Carolina Museum of Life and Science | Durham, NC | 8 | M | 1 | Mixed |
| 2062 | North Carolina Museum of Life and Science | Durham, NC | 4 | F | 1 | Mixed |
| 2247 | North Carolina Museum of Life and Science | Durham, NC | 8 months | M | 2 | Mixed |
| 2246 | North Carolina Museum of Life and Science | Durham, NC | 8 months | M | 1 | Mixed |
| 2079 | Northeastern Wisconsin Zoo | Green Bay, WI | 4 | M | 12 | Mixed |
| 2081 | Northeastern Wisconsin Zoo | Green Bay, WI | 4 | M | 1 | Mixed |
| 1931 | Point Defiance Zoo and Aquarium | Tacoma, WA | 6 | F | 1 | Kibble |
| 1935 | Point Defiance Zoo and Aquarium | Tacoma, WA | 6 | F | 1 | Whole meat |
| 1585 | Point Defiance Zoo and Aquarium | Tacoma, WA | 10 | F | 1 | Kibble |
| 2153 | Point Defiance Zoo and Aquarium | Tacoma, WA | 2 | F | 1 | Kibble |
| 1807 | Point Defiance Zoo and Aquarium | Tacoma, WA | 7 | F | 1 | Kibble |
| 1491 | Point Defiance Zoo and Aquarium | Tacoma, WA | 6 | M | 1 | Kibble |
| 1381 | Point Defiance Zoo and Aquarium | Tacoma, WA | 13 | M | 1 | Kibble |
| 1943 | Point Defiance Zoo and Aquarium | Tacoma, WA | 5 | M | 1 | Kibble |
| 1942 | Point Defiance Zoo and Aquarium | Tacoma, WA | 5 | M | 1 | Kibble |
| 1946 | Point Defiance Zoo and Aquarium | Tacoma, WA | 6 | F | 1 | Whole meat |
| 2003 | Point Defiance Zoo and Aquarium | Tacoma, WA | 5 | F | 1 | Kibble |
| 1416 | Point Defiance Zoo and Aquarium | Tacoma, WA | 13 | F | 1 | Kibble |
| 2078 | Point Defiance Zoo and Aquarium | Tacoma, WA | 3 | F | 1 | Kibble |
| 1363 | Point Defiance Zoo and Aquarium | Tacoma, WA | 14 | F | 1 | Kibble |
| 2139 | Point Defiance Zoo and Aquarium | Tacoma, WA | 2 | F | 1 | Kibble |
| 1861 | Point Defiance Zoo and Aquarium | Tacoma, WA | 6 | M | 1 | Kibble |
| 2007 | Point Defiance Zoo and Aquarium | Tacoma, WA | 5 | F | 1 | Kibble |
| 1496 | Point Defiance Zoo and Aquarium | Tacoma, WA | 11 | F | 1 | Kibble |
| 2138 | Point Defiance Zoo and Aquarium | Tacoma, WA | 2 | F | 1 | Kibble |
| 2076 | Point Defiance Zoo and Aquarium | Tacoma, WA | 4 | M | 1 | Kibble |
| 2132 | Point Defiance Zoo and Aquarium | Tacoma, WA | 2 | M | 1 | Kibble |
| 1927 | Point Defiance Zoo and Aquarium | Tacoma, WA | 6 | M | 1 | Kibble |
| 1415 | Point Defiance Zoo and Aquarium | Tacoma, WA | 13 | F | 1 | Kibble |
| 1928 | Point Defiance Zoo and Aquarium | Tacoma, WA | 5 | M | 1 | Kibble |
| 2077 | Point Defiance Zoo and Aquarium | Tacoma, WA | 4 | M | 1 | Kibble |
| 2006 | Point Defiance Zoo and Aquarium | Tacoma, WA | 5 | M | 1 | Kibble |
| 2005 | Point Defiance Zoo and Aquarium | Tacoma, WA | 5 | M | 1 | Kibble |
| 1382 | Point Defiance Zoo and Aquarium | Tacoma, WA | 13 | F | 1 | Kibble |
| 11600 | Sandy Ridge | Manteo, NC | 11 | F | 1 | Kibble |
| 11276 | Sandy Ridge | Manteo, NC | 15 | F | 1 | Mixed |
| 11599 | Sandy Ridge | Manteo, NC | 11 | F | 2 | Kibble |
| 11473 | Sandy Ridge | Manteo, NC | 12 | F | 1 | Kibble |
| 1922 | Trevor Zoo | Millbrook, NY | 5 | M | 1 | Kibble |
| 1479 | Trevor Zoo | Millbrook, NY | 11 | F | 1 | Kibble |
| 1932 | Wolf Haven International | Tenino, WA | 5 | F | 1 | Mixed |
